# Supplementary material for: Leveraging single-cell ATAC-seq and RNA-seq to identify disease-critical fetal and adult brain cell types
Source: Nat Commun. 2024 Jan 17;15:563. doi: 10.1038/s41467-024-44742-0 (PMC10794712; doi:10.1038/s41467-024-44742-0)
Supplement: Supplementary file 1 — Supplementary Information [file 41467_2024_44742_MOESM1_ESM.pdf]

Leveraging single-cell ATAC-seq and RNA-seq to identify disease-critical fetal and adult brain cell types

Supplementary figures

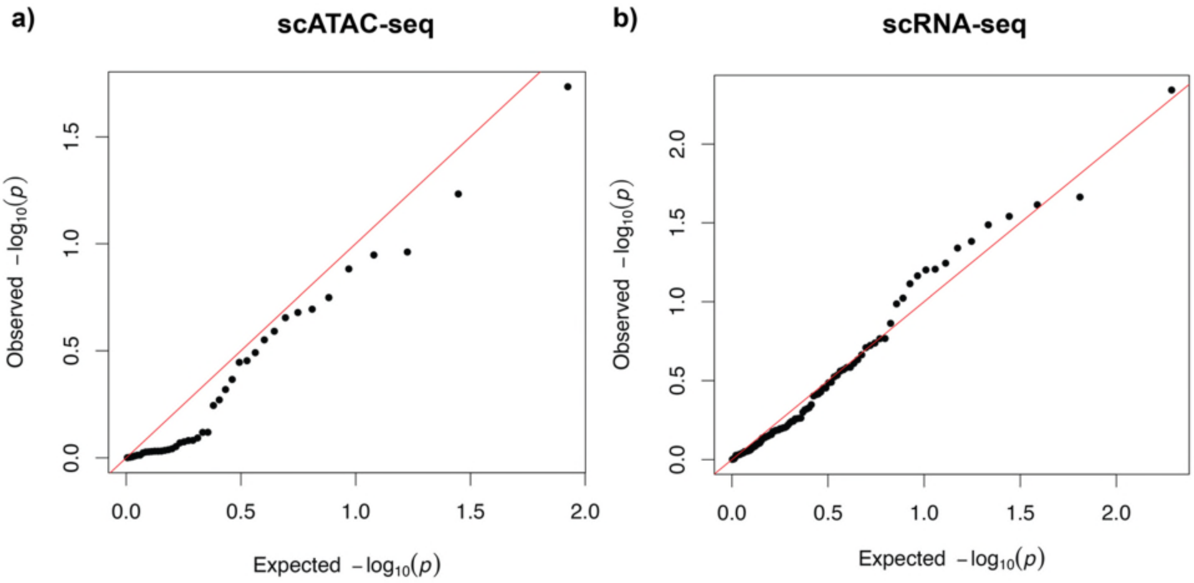

Figure S1. Distribution of P-values for nonzero τ^* in analyses of scATAC-seq and scRNA-seq fetal brain cell types across 6 non-brain-related diseases and complex traits. We report Q-Q plots for (a) scATAC-seq data and (b) scRNA-seq data. The Q-Q plots confirm a null distribution of P-values, validating the normality assumption of τ^* divided by its jackknife standard error. We used the S-LDSC to obtain $\tau*$ p-values (two-sided). We indicate significant results after adjusting for multiple comparisons based on per-dataset FDR < 5%.

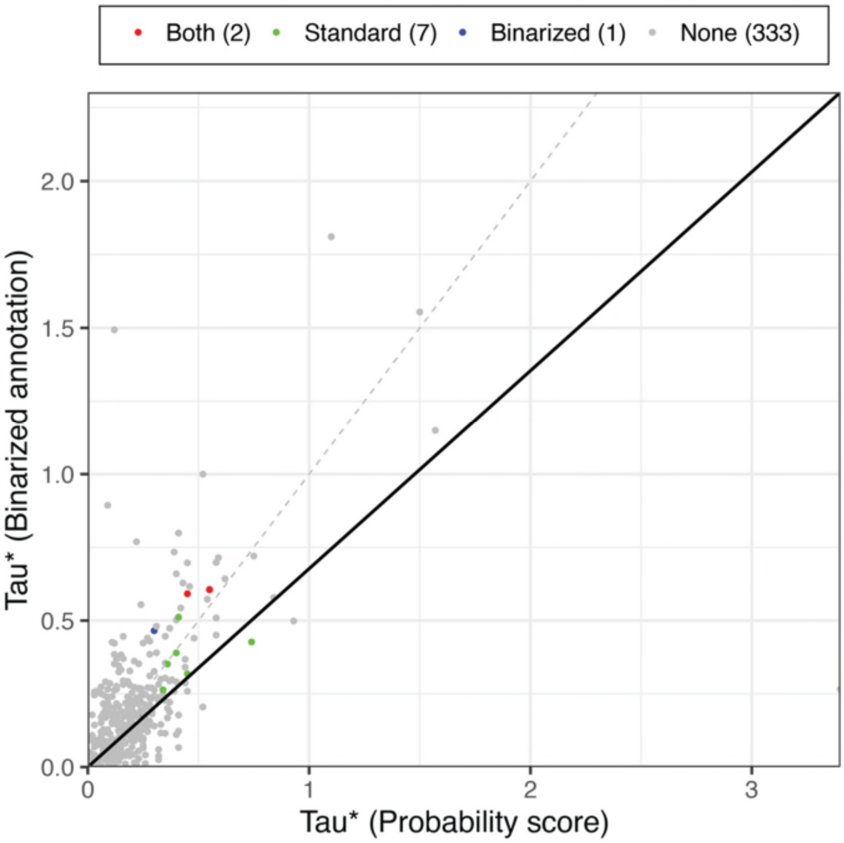

Figure S2. Comparison of scRNA-seq analysis using binarized annotations vs. primary analysis using probability scores. We report τ^* values for each analysis with fetal brain data. Dashed grey line denotes y=x. A regression of τ^* values using binarized annotations vs. τ^* values using probability scores yielded a slope of 0.677 (solid black line). There were 2 annotations that were significant in both analyses (red points), 7 annotations that were significant only using probability scores (green points), and 1 annotation that was significant only using binarized annotations (blue points).

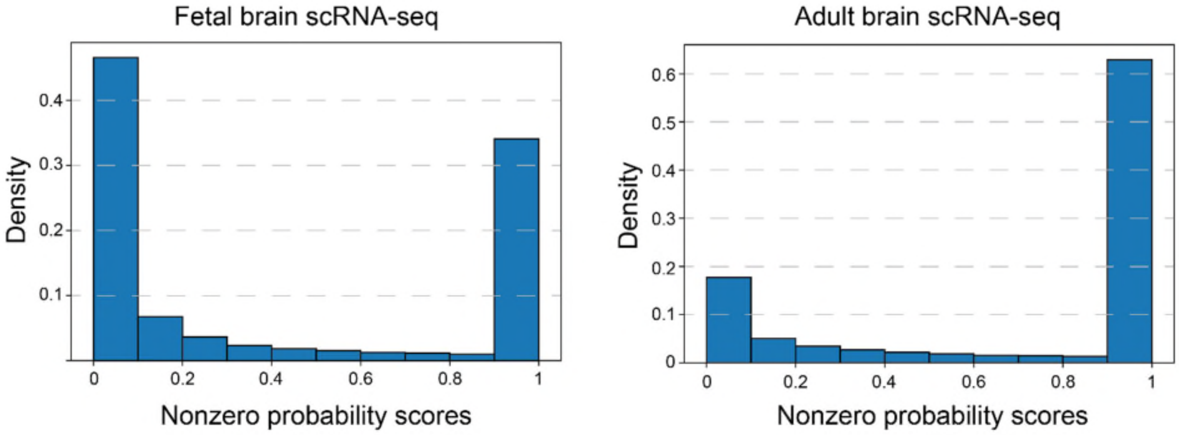

Figure S3. Distribution of nonzero probability scores for scRNA-seq data. We report the distribution of nonzero probability scores for fetal brain scRNA-seq data (left panel) and adult brain scRNA-seq data (right panel).

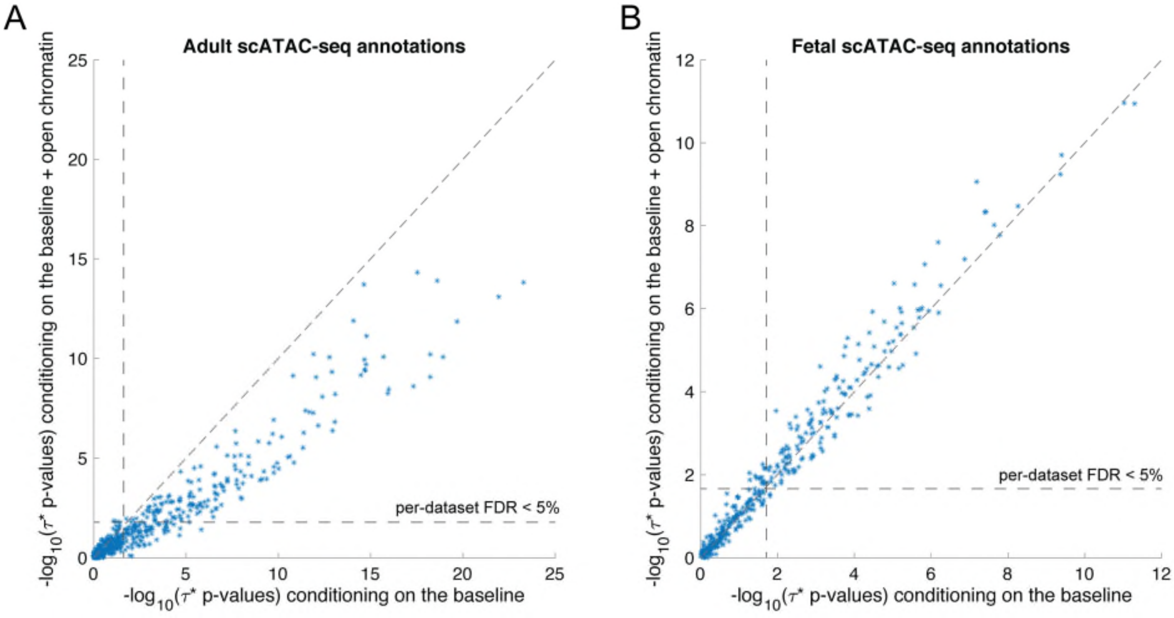

Figure S4. Disease enrichments of cell-type annotations derived from scATAC-seq data, conditioning on only baseline model. We report $-\log_{10}(\tau^*$ p-values) of (A) adult scATAC-seq and (B) fetal scATAC-seq cell-type annotations for two different models: (1) conditioning on only baseline model and (2) baseline model and the union of chromatin marks across cell types. Numeric results are found in Supplementary Data 22
