# Supplementary material for: Leveraging single-cell ATAC-seq and RNA-seq to identify disease-critical fetal and adult brain cell types
Source: Nat Commun. 2024 Jan 17;15:563. doi: 10.1038/s41467-024-44742-0 (PMC10794712; doi:10.1038/s41467-024-44742-0)
Supplement: Supplementary file 3 — Description of Additional Supplementary Files [file 41467_2024_44742_MOESM3_ESM.pdf]

Description of Additional Supplementary Files

File Name: Supplementary Data 1 - 25
Description:

Supplementary Data 1: List of 28 brain-related traits and 6 non-brain-related traits analyzed. For each trait, we report a trait identifier, trait description, reference, sample size, and heritability z-score.

Supplementary Data 2: Genetic correlation among 28 brain-related traits. We report the genetic correlation as estimated by S-LDSC across 28 GWAS summary association statistics.

Supplementary Data 3: Description of the baseline model. For each of 53 functional annotations in the baseline model, we provide the description, proportion of SNPs annotated, and reference.

Supplementary Data 4: Tissues and cell types analyzed. We summarize 83 cell types (for 4 single-cell datasets) and 26 tissues (for bulk chromatin data) analyzed.

Supplementary Data 5: Disease enrichments of cell-type annotations derived from fetal brain scATAC-seq data. For each of 392 trait-cell type pairs (28 brain traits * 14 cell types), we report the proportion of SNPs, heritability enrichments, and τ^*. We used the S-LDSC to obtain τ^* p-values (two-sided). We indicate significant results after adjusting for multiple comparisons based on per-dataset FDR < 5%.

Supplementary Data 6: Matched cell types across datasets. We report the matched cell types, defined as either exactly or closely matching cell types appearing in (A) fetal brain (scATAC-seq and scRNA-seq), (B) adult brain (scATAC-seq and scRNA-seq), (C) scATAC-seq (fetal and adult brain), and (D) scRNA-seq (fetal and adult brain).

Supplementary Data 7: Disease enrichments of cell-type annotations derived from fetal brain scRNA-seq data. For each of 952 trait-cell type pairs (28 brain traits * 34 cell types), we report the proportion of SNPs, heritability enrichments, and τ^*. We used the S-LDSC to obtain τ^* p-values (two-sided). We indicate significant results after adjusting for multiple comparisons based on per-dataset FDR < 5%.

Supplementary Data 8: Comparison between fetal brain scATAC-seq and fetal brain scRNA-seq cell-type annotations. For 13 cell types appearing in both fetal brain scATAC-seq and scRNA-seq datasets, we report correlation between two corresponding annotations and τ^* (conditioning on the baseline model, union of open chromatin regions, brain-specific enhancer-gene links of all genes, and to each other). We used the S-LDSC to obtain τ^* p-values (two-sided).

Supplementary Data 9: Correlation among cell-type annotations. We report the genome-wide Pearson correlation (*r*) across cell-type annotations from 4 single-cell datasets.

Supplementary Data 10: Disease enrichments of cell-type annotations derived from fetal brain scATAC-seq data in non-brain traits. For 84 trait-cell type pairs (6 non-brain traits * 14 cell types), we report the proportion of SNPs, heritability enrichments, and τ^*. We used the S-LDSC to obtain τ^* p-values (two-sided). We indicate significant results after adjusting for multiple comparisons based on per-dataset FDR < 5%.

Supplementary Data 11: Disease enrichments of cell-type annotations derived from fetal brain scRNA-seq data in non-brain traits. For 204 trait-cell type pairs (6 non-brain traits * 34 cell types), we report the proportion of SNPs, heritability enrichments, and τ^*. We used the S-LDSC to obtain τ^* p-values (two-sided). We indicate significant results after adjusting for multiple comparisons based on per-dataset FDR < 5%.

Supplementary Data 12: GREAT gene set enrichment of cell-type annotations derived from fetal brain datasets. For each cell-type annotation (A) from scATAC-seq and (B) from scRNA-seq, we report the top enriched gene ontology (GO) terms, fold enrichment, binomial test p-value, and FDR-corrected q-value. We report both unadjusted and adjusted p-values. Both p-values are based on a one-tailed binomial test from GREAT.

Supplementary Data 13: Disease enrichments of cell-type annotations derived from fetal brain scRNA-seq data (using window-based approach). We annotated SNPs +/- 100kb around genes, instead of brain-specific enhancer-gene links, and assessed the heritability enrichments. For each of 952 trait-cell type pairs (28 brain traits * 34 cell types), we report the proportion of SNPs, heritability enrichments, and τ^*. We used the S-LDSC to obtain τ^* p-values (two-sided). We indicate significant results after adjusting for multiple comparisons based on per-dataset FDR < 5%.

Supplementary Data 14: Disease-critical tissues using bulk chromatin data of fetal epigenomes. For each of 980 disease-tissue-chromatin mark triplets (28 brain traits * 5 fetal tissues * 7 chromatin marks), we report the proportion of SNPs, heritability enrichments, and τ^*. We considered 833 high-quality epigenomes9 and selected 25 fetal biosamples related to brain tissues. We considered tier 1 assays (DNase-seq, H3K4me1,

H3K4me3, H3K27ac, H3K36me3, H3K9me3 and H3K27me3), as most of observed data comes from tier 1

assays. Consistent with previous study[7,8,11], we built tissue annotations by annotating chromatin peaks using the MACS2[28]. We used the S-LDSC to obtain τ^* p-values (two-sided). We indicate significant results after adjusting for multiple comparisons based on per-dataset FDR < 5%.

Supplementary Data 15: Disease enrichments of cell-type annotations derived from adult brain scATAC-seq data. For each of 504 trait-cell type pairs (28 brain traits * 18 cell types), we report the proportion of SNPs, heritability enrichments, and τ^*. We used the S-LDSC to obtain τ^* p-values (two-sided). We indicate significant results after adjusting for multiple comparisons based on per-dataset FDR < 5%.

Supplementary Data 16: Disease enrichments of cell-type annotations derived from adult brain scRNA-seq data. For each of 476 trait-cell type pairs (28 brain traits * 17 cell types), we report the proportion of SNPs, heritability enrichments, and τ^*. We used the S-LDSC to obtain τ^* p-values (two-sided). We indicate significant results after adjusting for multiple comparisons based on per-dataset FDR < 5%.

Supplementary Data 17: Comparison between adult brain scATAC-seq and adult brain scRNA-seq annotations. For 6 cell types appearing in both adult brain scATAC-seq and scRNA-seq datasets, we report correlation between two corresponding annotations and τ^* (conditioning on the baseline model, union of open chromatin regions, brain-specific enhancer-gene links of all genes, and to each other). We used the S-LDSC to obtain τ^* p-values (two-sided).

Supplementary Data 18: Comparison between fetal brain scATAC-seq and adult brain scATAC-seq annotations. For 3 cell types appearing in both fetal brain scATAC-seq and adult brain scATAC-seq datasets, we report correlation between two corresponding annotations and τ^* (conditioning on the baseline model, union of open chromatin regions, brain-specific enhancer-gene links of all genes, and to each other). We used the S-LDSC to obtain τ^* p-values (two-sided).

Supplementary Data 19: Comparison between fetal brain scRNA-seq and adult brain scRNA-seq annotations. For 6 cell types appearing in both fetal brain scRNA-seq and adult brain scRNA-seq datasets, we report correlation between two corresponding annotations and τ^* (conditioning on the baseline model, brain-specific enhancer-gene links of all genes, and to each other). We used the S-LDSC to obtain τ^* p-values (two-sided).

Supplementary Data 20: Disease enrichments of cell-type annotations derived from adult brain scATAC-seq data in non-brain traits. For 108 trait-cell type pairs (6 non-brain traits * 18 cell types), we report the proportion of SNPs, heritability enrichments, and τ^*. We used the S-LDSC to obtain τ^* p-values (two-sided). We indicate significant results after adjusting for multiple comparisons based on per-dataset FDR < 5%.

Supplementary Data 21. Disease enrichments of cell-type annotations derived from adult brain scRNA-seq data in non-brain traits. For 102 trait-cell type pairs (6 non-brain traits * 17 cell types), we report the proportion of SNPs, heritability enrichments, and τ^*. We used the S-LDSC to obtain τ^* p-values (two-sided). We indicate significant results after adjusting for multiple comparisons based on per-dataset FDR < 5%.

Supplementary Data 22. Disease enrichments of cell-type annotations derived from scATAC-seq data, conditioning on only baseline model. We assessed heritability enrichments of (A) adult scATAC-seq and (B) fetal scATAC-seq cell-type annotations, conditioning on only baseline model (instead of conditioning on the baseline model and the union of chromatin marks across cell types). We report the proportion of SNPs, heritability enrichments, and τ^*. We used the S-

LDSC to obtain τ^* p-values (two-sided). We indicate significant results after adjusting for multiple comparisons based on per-dataset FDR < 5%.

Supplementary Data 23. GREAT gene set enrichment of cell-type annotations derived from adult brain datasets. For each cell-type annotation (A) from scATAC-seq and (B) from scRNA-seq, we report the top enriched gene ontology (GO) terms, fold enrichment, binomial test p-value, and FDR-corrected q-value. We report both unadjusted and adjusted p-values. Both p-values are based on a one-tailed binomial test from GREAT.

Supplementary Data 24. Disease enrichments of cell-type annotations derived from adult brain scRNA-seq data (using window-based approach). We annotated SNPs +/- 100kb around genes, instead of brain-specific enhancer-gene links, and assessed the heritability enrichments. For each of 476 trait-cell type pairs (28 brain traits * 17 cell types), we report the proportion of SNPs, heritability enrichments, and τ^*. We used the S-LDSC to obtain τ^* p-values (two-sided). We indicate significant results after adjusting for multiple comparisons based on per-dataset FDR < 5%.

Supplementary Data 25. Disease-critical tissues using bulk chromatin data of adult epigenomes. For each of 4,116 disease-tissue-chromatin mark triplets (28 brain traits * 21 adult tissues * 7 chromatin marks), we report the proportion of SNPs, heritability enrichments, and τ^*. We considered 32 adult biosamples related to brain tissues and tier 1 assays (DNase-seq, H3K4me1, H3K4me3, H3K27ac, H3K36me3, H3K9me3 and H3K27me3). Donor information can be found in [9]. We used the S-LDSC to obtain τ^* p-values (two-sided). We indicate significant results after adjusting for multiple comparisons based on per-dataset FDR < 5%.
