# Supplementary material for: Leveraging single-cell ATAC-seq and RNA-seq to identify disease-critical fetal and adult brain cell types
Source: Nat Commun. 2024 Jan 17;15:563. doi: 10.1038/s41467-024-44742-0 (PMC10794712; doi:10.1038/s41467-024-44742-0)
Supplement: Supplementary file 5 — Reporting Summary [file 41467_2024_44742_MOESM5_ESM.pdf]

nature portfolio

Corresponding author(s): Samuel Kim, Buu Truong, Alkes Price

Last updated by author(s): Aug 16, 2023

Reporting Summary

Nature Portfolio wishes to improve the reproducibility of the work that we publish. This form provides structure for consistency and transparency in reporting. For further information on Nature Portfolio policies, see our Editorial Policies and the Editorial Policy Checklist.

Statistics

For all statistical analyses, confirm that the following items are present in the figure legend, table legend, main text, or Methods section.

n/a	Confirmed	
☐	☒	The exact sample size (*n*) for each experimental group/condition, given as a discrete number and unit of measurement
☐	☒	A statement on whether measurements were taken from distinct samples or whether the same sample was measured repeatedly
☐	☒	The statistical test(s) used AND whether they are one- or two-sided<br>*Only common tests should be described solely by name; describe more complex techniques in the Methods section.*
☐	☒	A description of all covariates tested
☐	☒	A description of any assumptions or corrections, such as tests of normality and adjustment for multiple comparisons
☐	☒	A full description of the statistical parameters including central tendency (e.g. means) or other basic estimates (e.g. regression coefficient) AND variation (e.g. standard deviation) or associated estimates of uncertainty (e.g. confidence intervals)
☐	☒	For null hypothesis testing, the test statistic (e.g. *F*, *t*, *r*) with confidence intervals, effect sizes, degrees of freedom and *P* value noted<br>*Give P values as exact values whenever suitable.*
☒	☐	For Bayesian analysis, information on the choice of priors and Markov chain Monte Carlo settings
☒	☐	For hierarchical and complex designs, identification of the appropriate level for tests and full reporting of outcomes
☐	☒	Estimates of effect sizes (e.g. Cohen's *d*, Pearson's *r*), indicating how they were calculated

Our web collection on statistics for biologists contains articles on many of the points above.

Software and code

Policy information about availability of computer code

Data collection	The GWAS data used in this data set is public available and the single cell ATAC seq is obtained from Domcke et al. Science 2020, Corces et al. Nature Genetics, 20220 and scRNAseq is from Cao et al. Science 2020, Velmeshev et al. Science 2019.
Data analysis	The cell-type annotations and source code for primary analyses are available at https://alkesg roup.broadinstitute.org/LDSCORE/Kim_ATAC/.<br>S-LDSC software: https://github.com/bulik/ldsc<br>GREAT (Genomic Regions Enrichment of Annotations Tool): http://great.stanford.edu/

For manuscripts utilizing custom algorithms or software that are central to the research but not yet described in published literature, software must be made available to editors and reviewers. We strongly encourage code deposition in a community repository (e.g. GitHub). See the Nature Portfolio guidelines for submitting code & software for further information.

Data

Policy information about availability of data

All manuscripts must include a data availability statement. This statement should provide the following information, where applicable:

- Accession codes, unique identifiers, or web links for publicly available datasets
- A description of any restrictions on data availability
- For clinical datasets or third party data, please ensure that the statement adheres to our policy

GWAS summary statistics: https://alkesgroup.broadinstitute.org/sumstats_formatted/

Generated cell-type annotations: https://alkesgroup.broadinstitute.org/LDSCORE/Kim_AT AC/
Domcke et al. fetal scATAC-seq data: https://atlas.brotmanbaty.org/bbi/human-chromatin -during-development/
Cao et al. fetal scRNA-seq data: https://atlas.brotmanbaty.org/bbi/human-gene-express ion-during-development/
Corces et al. scATAC-seq data: http://epigenomegateway.wustl.edu/legacy/?genome=hg38
&session=drS3o1n4kJ
Velmeshev et al. scRNA-seq data: https://autism.cells.ucsc.edu/ baseline (v.1.2) annotations: https://data.broadinstitute.org/alkesgroup/LDSCORE/
1000 Genomes Project Phase 3 data: ftp://ftp.1000genomes.ebi.ac.uk/vol1/ftp/release/20130502

Research involving human participants, their data, or biological material

Policy information about studies with human participants or human data. See also policy information about sex, gender (identity/presentation), and sexual orientation and race, ethnicity and racism.

Reporting on sex and gender	The data was not selective on sex and gender
Reporting on race, ethnicity, or other socially relevant groupings	The data was not selective on race, ethnicity or any social group
Population characteristics	The study conducted the analyses with public available data with fetal and adult cell types.
Recruitment	The GWAS data is public available and individuals recruited based on consent in each data
Ethics oversight	All individuals in the UK Biobank inclued in the analyses were approved under application 16549.

Note that full information on the approval of the study protocol must also be provided in the manuscript.

Field-specific reporting

Please select the one below that is the best fit for your research. If you are not sure, read the appropriate sections before making your selection.

☒ Life sciences ☐ Behavioural & social sciences ☐ Ecological, evolutionary & environmental sciences

For a reference copy of the document with all sections, see nature.com/documents/nr-reporting-summary-flat.pdf

Life sciences study design

All studies must disclose on these points even when the disclosure is negative.

Sample size	We analyzed 28 distinct brain-related diseases and traits, 146 sets of GWAS summary association statistics, including 83 traits from the UK Biobank and 63 traits from publicly available sources, with z-scores for total SNP-heritability of at least 6
Data exclusions	Data exclusion was not relevant
Replication	The analyses were compared between fetal data and adult data.
Randomization	Randomization was not relevant
Blinding	Blinding is not relevant in our study, because the data collected by the biobanks and the ID of participants were already encrypted

Reporting for specific materials, systems and methods

We require information from authors about some types of materials, experimental systems and methods used in many studies. Here, indicate whether each material, system or method listed is relevant to your study. If you are not sure if a list item applies to your research, read the appropriate section before selecting a response.

Materials & experimental systems

n/a	Involved in the study	
☒	☐	Antibodies
☒	☐	Eukaryotic cell lines
☒	☐	Palaeontology and archaeology
☒	☐	Animals and other organisms
☒	☐	Clinical data
☒	☐	Dual use research of concern
☒	☐	Plants

Methods

n/a	Involved in the study	
☒	☐	ChIP-seq
☒	☐	Flow cytometry
☒	☐	MRI-based neuroimaging
